# Decentralised training for medical students: a scoping review

**DOI:** 10.1186/s12909-017-1050-9

**Published:** 2017-11-09

**Authors:** Marietjie de Villiers, Susan van Schalkwyk, Julia Blitz, Ian Couper, Kalavani Moodley, Zohray Talib, Taryn Young

**Affiliations:** 1 Division of Family Medicine and Primary Care, Faculty of Medicine and Health Sciences, Stellenbosch University, Stellenbosch, South Africa; 2 Centre for Health Professions Education, Faculty of Medicine and Health Sciences, Stellenbosch University, Stellenbosch, South Africa; 3 Ukwanda Centre for Rural Health, Faculty of Medicine and Health Sciences, Stellenbosch University, Stellenbosch, South Africa; 4 Centre for Evidence-based Health Care, Faculty of Medicine and Health Sciences, Stellenbosch University, Stellenbosch, South Africa; 5 Departments of Medicine and Health Policy, George Washington University, Washington DC, USA

**Keywords:** Decentralised training, Distributed, Rural, Medical student, Undergraduate

## Abstract

**Background:**

Increasingly, medical students are trained at sites away from the tertiary academic health centre. A growing body of literature identifies the benefits of decentralised clinical training for students, the health services and the community. A scoping review was done to identify approaches to decentralised training, how these have been implemented and what the outcomes of these approaches have been in an effort to provide a knowledge base towards developing a model for decentralised training for undergraduate medical students in lower and middle-income countries (LMICs).

**Methods:**

Using a comprehensive search strategy, the following databases were searched, namely EBSCO Host, ERIC, HRH Global Resources, Index Medicus, MEDLINE and WHO Repository, generating 3383 references. The review team identified 288 key additional records from other sources. Using prespecified eligibility criteria, the publications were screened through several rounds. Variables for the data-charting process were developed, and the data were entered into a custom-made online Smartsheet database. The data were analysed qualitatively and quantitatively.

**Results:**

One hundred and five articles were included. Terminology most commonly used to describe decentralised training included ‘rural’, ‘community based’ and ‘longitudinal rural’. The publications largely originated from Australia, the United States of America (USA), Canada and South Africa. Fifty-five percent described decentralised training rotations for periods of more than six months. Thematic analysis of the literature on practice in decentralised medical training identified four themes, each with a number of subthemes. These themes were student learning, the training environment, the role of the community, and leadership and governance.

**Conclusions:**

Evident from our findings are the multiplicity and interconnectedness of factors that characterise approaches to decentralised training. The student experience is nested within a particular context that is framed by the leadership and governance that direct it, and the site and the community in which the training is happening. Each decentralised site is seen to have its own dynamic that may foreground certain elements, responding differently to enabling student learning and influencing the student experience. The insights that have been established through this review have relevance in informing the further expansion of decentralised clinical training, including in LMIC contexts.

## Background

The clinical training of medical students is an essential part of the curriculum and has traditionally occurred in large academic health centres, located close to medical schools. An increasing trend in clinical training is to allocate students to sites (urban, peri-urban and rural) away from the tertiary health care centre [1]. The challenges of increasing numbers have been a factor in this shift, but there are many other imperatives, including responding to a desire to increase student exposure to the breadth of the health care system, the burden of disease and the social determinants of health. In particular, there has been a focus on training students in communities, often in rural contexts [2, 3]. Decentralised training in rural areas has a strong workforce imperative, based on evidence that training students from and in such areas increases the likelihood of rural practice [4]. Social responsibility has also long been a driving factor behind community-based health professions education, with the knowledge that placing students in communities will provide both hands-on understanding of the problems that they will face in their future practice and the skills for addressing these while contributing to the quality of life in a particular community [5]. Increasingly, such initiatives are being driven by a realisation of the academic value of such decentralised training in terms of the exposure of students to generalist care of patients with undifferentiated problems. It provides broader exposure for students to a range of patients in terms of the ecology of medical care [6] and can enhance their training for the roles that they may be called upon to play as graduates in responding to the health care needs of a population [7].

Placing students at decentralised sites requires effort and resources on the part of the educational institution, the health services, the training site, the community within which students are placed and the clinicians who take up the responsibility of training [8–10]. In addition, there are many issues relating to the curriculum and, in particular, assessment of students while on this decentralised platform. The literature suggests that students are not academically disadvantaged by being trained at smaller rural and remote sites [11, 12] and that in many instances, students believe that they have a more meaningful learning experience than they expected to receive in urban tertiary hospital settings [2, 13]. They also develop a more complex sense of professional identity [14] and feel more prepared to become doctors [15]. They are often advantaged academically [16], with one study reporting that the rural cohort advanced better academically compared to their peers [13].

There is an increasing body of literature that describes and evaluates clinical training at decentralised sites from many parts of the world, predominantly Australia, North America and, more recently, South Africa. We therefore undertook a scoping review of the prevailing literature relating to the current status of decentralised training for undergraduate medical students. Specifically, we wanted to determine trends and discern what factors characterised current approaches to decentralised training. The intention was that this review would provide us with a knowledge base that would support our ongoing work towards developing a model for decentralised training for undergraduate medical students in LMICs.

## Methods

Scoping reviews are more recent entrants into the suite of review methodologies, and their aim is to hone in on the key features of a particular issue or concept as these can be gleaned from relevant literature [17]. Typically, scoping reviews generate descriptive narratives that represent a synthesis of the primary and other sources of evidence that are available. A recent synthesis of the work of Arksey and O’Malley, and Levac et al. [18, 19] proposed a list of six stages for those undertaking a scoping study. We aligned our methods with the first five of these stages. After the team had been assembled, we confirmed the purpose of the study and co-operatively developed a plan to guide the review (Steps 1 and 2). The specific research questions were as follows:

- What decentralised models currently exist for the training of undergraduate medical students? (What has been/is being done?)
- How have these models been implemented? (What approaches have been adopted?)
- What have been the results of these approaches? (What has happened as a result of implementing these approaches?)

### Criteria for considering studies for inclusion and exclusion (step 3)

Through an iterative process that included regular team meetings, it was decided that the review would consider all decentralised training activities for undergraduate medical students that were described in the literature from all sites that were removed from the central academic training hospital(s) (rural sites, primary clinics, district hospitals, regional hospitals, etc.). The outcomes considered included educational outcomes (transformative learning, culture of learning and retention), community outcomes (social accountability), patient outcomes (patient satisfaction), staff outcomes (retention, resilience, job satisfaction and learning), organisational outcomes (culture of learning), health service outcomes (quality of care and health systems strengthened), costing and cost-effectiveness, and relationship between the decentralised site and the central (referral) health service. To ensure feasibility, only studies published between January 2005 and December 2015 that were available in English were included.

The following databases were searched in July 2015 using a comprehensive search strategy (Table 1): EBSCO Host, ERIC, HRH Global Resources, Index Medicus, MEDLINE and WHO Repository. This generated 3383 references. The review team also identified key additional literature in the field that had not been uncovered by the search.

**Table 1:** Search strategy

1. ‘Physicians’[Mesh] OR physician* OR ‘medical doctor*’ OR ‘general practitioner*’ OR GPs title, abstract
2. Training OR teaching OR ‘education* program*’ or curriculum [title, abstract]
3. 1 and 2
4. ‘Education, Medical, Undergraduate’[Mesh])
5. 3 or 4
6. decentraliz* OR decentralis* OR distributed OR ‘community-based’ OR ‘community-engaged’ OR ‘on the job’ OR ‘in service’ OR rural OR extramural [Title/Abstract]
7. 5 and 6

### Study selection, data collection and interpretation (steps 4 and 5)

Using the prespecified eligibility criteria, the first round of review created a shortlist by screening each publication’s abstract to eliminate articles that were not in the scope of this review. The resulting list was checked and peer-reviewed by a second member of the team. The full text of all shortlisted publications was obtained, entered in an Endnote database and reviewed independently by two reviewers. Disagreements were resolved through discussion.

A range of variables that would comprise the data-charting form was developed by the team through an iterative and consultative process (Table 2). Data were then extracted from the included literature and entered into a database (Smartsheet) that allowed for multiple online users (a link to the database is given under Declarations).

**Table 2:** Categories extracted from included literature

1. Cadre being educated (undergraduate medical or undergraduate medical plus).
2. Description of facility (community, clinic or district hospital).
3. Location of site (country, town or region).
4. Is the site rural, peri-urban or urban?
5. How much time do students spend at the site?
6. Is reference made to the evaluation process of the intervention?
7. Who/what was evaluated (students, staff or curricula)?
8. Description of the intervention.
9. Aim of the intervention.
10. Critical elements of the process of intervention.
11. Indicators used to measure the success of the programme.
12. Level of success documented for the site.
13. Success factors for establishing a training site.
14. Relevance for establishing a model for training.

The included studies were analysed quantitatively and qualitatively (Step 5). During the quantitative analysis, numerical summaries of type of article, duration of rotation, rural-urban mix, countries and facilities where the training was done, whether evaluation was conducted and focus of the evaluation were generated. The numbers for these were captured and analysed using Smartsheet.

Three categories in the data chart generated descriptive data that related to: level of success documented for this site, success factors for establishing a training site and relevance for establishing a model for training (numbers 12-14 in Table 2). As our intention was both to describe and understand the nature and extent of these variables, we subjected this data to content analysis, which was applied as follows [20]. Initial coding was done by MdV and SvS looking for emergent patterns across the data. The resultant code lists were reviewed and synthesised by JB, ZT and SvS. Discrepancies were resolved through discussion among team members. The codes were then grouped into higher order themes and subthemes by SvS, JB, and ZT, which were subsequently reviewed by the entire team. Finally, MdV again searched across all 105 publications to ensure accurate representation of the included studies across all the themes and subthemes. Thomas et al. [21] suggest an optional sixth step in the scoping review process, namely that of consultation with key stakeholders around the outcomes of the review. It is our intention to facilitate such consultation as part of a larger project within which this review is located.

Ethics approval was obtained from the Stellenbosch University Human Research Ethics Committee, approval number #N16/03/034.

## Results

### Description of studies

Using the eligibility criteria and a peer-review process, 105 articles were ultimately included in the analysis (listed in the Smartsheet database).

**Fig. 1:**
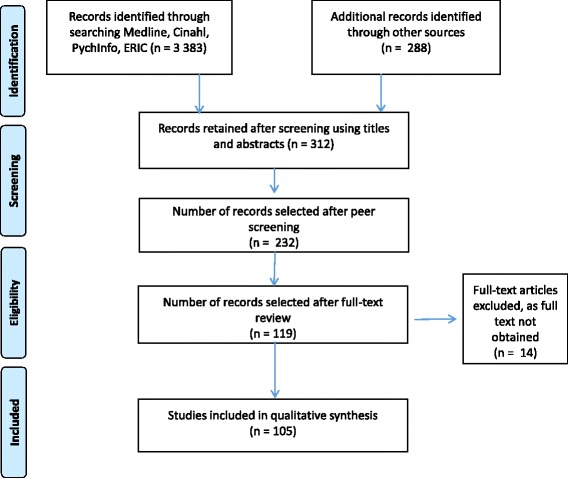
Study identification and selection

Figure 1 shows the outcome of the study identification and selection process.

In 24 of the publications, we found literature related to decentralised training in the form of systematic reviews, World Health Organization and other policy reports, various other reviews, Association for Medical Education in Europe and Best Evidence Medical Education guides, book chapters, meeting reports, short reports and commentaries. In 70% (*n* = 63) of the remaining 81 articles, the authors described an evaluation of their decentralised training intervention. In 42% (*n* = 34) of these 63 studies, the evaluation focussed on students only. In a further 12% of studies (*n* = 10), students as well as a combination of other role players, including patients, communities, clinicians, faculty, hospital staff and so forth, were the focus of the evaluation. Table 3 shows the focus of the evaluation for these studies.

**Table 3:** Focus of the evaluation

Focus of the evaluation	Number of studies
Students	34
Students AND various others^a^	10
Clinical supervisors/preceptors/site facilitators	4
Doctors in community/rural doctors	4
Communities	3
Faculty (staff)/school	2
Student projects	2
Site facilitators AND community and patients	1
Costing	1
Graduates	1
Health outcomes	1
Total	63

^a^Various others included educators, preceptors, faculty, staff, clinicians, managers, community representatives, general practitioners, patients and community educators

The length of the decentralised rotations was documented in 47 articles. Fifty-five percent of these (*n* = 26) described decentralised training rotations for periods of more than six months, classified by the authors as long term, 34% (*n* = 16) of the articles described medium-term exposures (1-6 months) and 10% (*n* = 5) of the articles reviewed specified rotations of less than a month.

In the 58 articles in which the physical placement of the rotation was specified, 38 (65,5%) were described as rural, 4 (7%) as urban and 16 (27,5%) as both rural and urban.

**Fig. 2:**
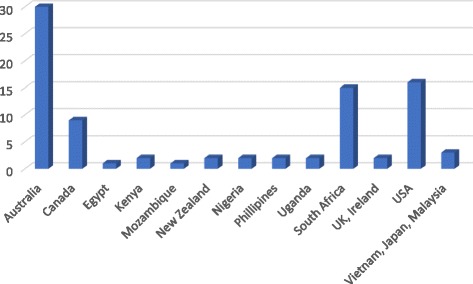
Number of publications by country

Most of the publications reported work conducted in only one country, with five studies involving two or three multi-country sites (*n* = 87) (Fig. 2). The source of publication generally reflected the spread of countries where the training was taking place.

Decentralised training was reported as being conducted across a range of facilities. In total, 121 facilities were counted (Table 4).

**Table 4:** Facilities where decentralised training was being conducted

Facility	Number
Community	34
District/local/rural hospital	31
Clinic	18
General practice	12
Distributed campus	9
Regional hospital	8
Community health centres	6
Rehabilitation service	2
Schools	1
Total	121

### Description of themes

A key feature of this review was maintaining definitional clarity. We found that the words that were most commonly used in the included studies to describe medical training that happened away from the tertiary hospital were ‘rural’, ‘community based’ and ‘longitudinal rural’. Other terms less frequently used included ‘regionalised’, ‘decentralised’, ‘distributed’ and ‘peripheral’. Terms such as ‘remote’, ‘field teaching’, ‘nonacademic’, ‘district health’ and ‘bush learning’ were also sometimes used. This emphasises the diversity in this area, and the findings described below need to be seen in this context. In addition, the authors saw decentralised training and the resultant student learning as directly linked, with the latter as based on the intended outcomes of the training.

The literature on current practice and approaches in decentralised medical training as captured in the included studies can be categorised into four broad themes:

- Student learning
- Training environment
- Role of community
- Leadership and governance

Table 5 lists the themes and the subthemes.

**Table 5:** Themes and subthemes

Theme	Subtheme
Student learning	Student selection
	Learning experience
	Curriculum implications
	Learning outcomes
	Assessment
Training environment	Environment
	Infrastructure
	Clinician supervisors
Role of community	Community immersion
	Community partnerships
	Social accountability
Leadership and governance	Visionary leadership
	Stakeholder engagement
	Funding
	Evaluation

### Student learning

Various aspects of the student experience at decentralised sites emerged as themes from the literature. These include student selection, the learning experience, curriculum implications, learning outcomes and assessment.

#### Student selection

Well-considered student selection was considered an essential element of a decentralised training programme [22–25] with calls such as ‘select students wisely’ and ‘admit the right student’. In this regard, most articles pointed to the evidence about rural background as a proxy for improved rural recruitment and retention [23, 26–32]. Students with an urban background who were motivated to learn and live in decentralised areas were also inclined to stay after graduation [33, 34]. In addition to academic criteria, student selection should also be based on personal attributes such as motivation and interpersonal and communication skills [35].

#### Learning experience

The literature found that decentralised training exposed students to everyday situations and a case load relevant to the needs of the community – an exposure quite different from that at the tertiary teaching complex [36–38]. Students learnt a holistic appreciation of medicine by experiencing undifferentiated and comprehensive care [1, 39]. Hands-on experience with more patients enhanced their clinical, procedural and community health skills [31, 40–43]. There was less competition for learning opportunities, given the smaller numbers that were typically found at these sites, and the students were exposed to co-operative approaches as they participated as members of the health care team [30, 40, 44] in various contexts. This facilitated the development of their cultural and ethical competencies [45–47].

#### Curriculum implications

There was a strong indication by the included studies that longer and longitudinal rotations, whereby students were immersed in the context, were more beneficial for clinical learning [13, 14, 22, 26, 28, 29, 33, 44, 46, 48–53]. This facilitated continuity in terms of patients and the community, the preceptors and the learning environment [51, 54] and fostered the adoption of an integrated approach to learning [50, 55]. Scheduling of rotations needed to move to longer and longitudinal exposure, especially from the start of the clinical years [32, 56].

According to the studies, decentralised training required ongoing curriculum renewal. The curriculum had to be flexible, responsive to community needs and underpinned by the principles of social accountability [57–59]. The timing of the first exposure and how the theoretical input linked with the practical training were important [28]. An integrated (clinical and public health) approach, educational continuity and equivalence in multiple settings were seen as important [52, 53, 60, 61]. A need for interprofessional learning to be imbedded in the curriculum [3, 57, 62, 63] as well as opportunities for developing ethical and cultural competencies was also identified [64].

#### Learning outcomes

The performance of students in decentralised training settings was reported to be either similar to or better than those following the ‘standard’ curriculum [24, 29, 31, 55, 65–67]. Students had a better understanding of decentralised training, valued training in decentralised areas and were more committed to rural and primary care practice after their decentralised training rotations [25, 27, 35, 43, 68]. More graduates who had trained in decentralised areas returned to these areas to practise than did their counterparts who had studied at the tertiary hospital [1, 27, 32, 67, 69].

Students were assessed to have improved practical skills after decentralised training [1, 41, 70–72], resulting in higher confidence levels [1, 73, 74]. The decentralised environment also facilitated their adoption of working in an interprofessional team [44, 62, 75]. Evidence was found of changes in behaviours and attitudes with the adoption of a professional approach to their practice [64, 76].

#### Assessment

A few articles commented on optimal assessment in decentralised training, including that it should be based on outcome assessment [77], be kept simple [78], use multiple assessment tools [71] and benchmark the content against the parallel curriculum at the tertiary centre [67].

### Training environment

The context within which the training occurs is critical in further framing the student’s learning experience. Thus, the second theme foregrounds the environment and then focusses on physical infrastructure on the one hand and those people who shape the student’s learning experience on the other hand.

#### Environment

Environmental issues were found to be key to the success of decentralised clinical placements [79]. All decentralised sites had their own strengths and weaknesses in which the context of the site played an important role [39, 50, 80]. Considerations in selecting decentralised training sites included the physical environment, the training environment, tutor characteristics, patient involvement and university responsibilities [74]. Well-functioning health care facilities were well suited to being decentralised training sites [45, 70] as they supported a mindset shift to incorporate a teaching role [38].

#### Infrastructure

Adequate physical infrastructure and space to support teaching and learning were seen as necessary [38, 49, 69, 74, 79, 81]. Providing adequate student accommodation at decentralised sites was a challenge [37, 67, 81–83]. Interactive communicative technology equipment and connectivity was essential for internet access, teaching on a web-based platform and online access to learning resources such as libraries [29, 38, 66, 67, 74, 77, 84–87]. Technology assisted in alleviating isolation at decentralised sites [85, 88].

#### Clinician supervisors

The availability of human resources was found to play an important role in decentralised training [38, 50, 59, 74, 84]. This included not only the clinician supervisors but also other members of the health care team at the facility [38, 74]. The review highlighted the need to recruit clinician supervisors (we are using this term as equivalent to preceptors, educators etc.) who are willing, committed and motivated to train the next generation of decentralised practitioners [14, 23, 37, 89–92]. Supervisors, however, needed orientation, information and training to be ready for the task [29, 57, 67, 72, 93, 94]. Academic programme faculty had to be involved with and supportive of the clinician supervisors [9, 22, 41, 95–97]. Continuity of supervision was seen as important as it facilitated the development of relationships between students and their supervisors and reinforced positive role models [2, 26, 39, 50, 54].

Benefits for supervisors included enjoyment of teaching, positive impact of the students, greater job satisfaction, workforce retention and professional development [44, 80, 83, 98]. Some articles spoke of the supervisor’s new role as a teacher providing ‘new meaning’ to his/her practice [50]. Consideration had to be given to awarding faculty status and incentives to these supervisors [69, 99]. Challenges in staffing included workforce shortages and human resources constraints [49, 84]. It was therefore important that optimal student-supervisor ratios be considered for the particular context [100, 101].

### Role of community

Student exposure to the breadth of the health care system implies an engagement at community level. The theme explores this engagement in greater depth.

#### Community immersion

The benefits for a range of stakeholders when clinical training takes place involving immersion in local communities were described in a number of the included studies. These benefits included addressing health workforce issues, changing attitudes and perceptions of students, faculty and community, rich real-life training experiences, closer relationships between faculty and community, and a positive impact on community health outcomes [12, 39, 61, 81, 102–107]. The challenges of community immersion, however, emerged from poor communication between faculty and community, language barriers and a lack of cultural and religious sensitivity [108]. A common vision, buy-in from stakeholders and commitment of all parties were listed as being important [39, 73].

#### Community partnerships

Strong partnerships with communities were described as a characteristic of successful decentralised training and were seen as an important prerequisite for scaling up such interventions [29, 53, 77]. These partnerships should be based on collaboration, active community involvement and in-depth engagement with the community about its context and health needs [22, 26, 93, 95, 109–111]. Community boards representing (amongst others) health services, community organisations, local leaders and the medical school fostered involvement of the community at large [107].

#### Social accountability

Lastly, the studies indicated that decentralised training should meet the needs of local communities and contribute to the improvement of health outcomes [22, 46, 59, 95, 106, 110, 111]. This social accountability mandate, “matching curriculum to cause and context” [59], was seen as very important for reciprocity, leading to shared ownership of the educational endeavour [111]. Despite this being such a strong recommendation from the literature, a systematic review found that medical schools did not as a rule involve communities in identifying the health priorities of the community [95].

### Leadership and governance

Moving an academic endeavour away from the institutional core requires visionary leadership. At the same time, care needs to be taken to ensure good governance, often at a distance.

#### Visionary leadership

Many of the included studies highlighted the importance of visionary leadership from management and academic staff at the training institutions to drive the implementation and upscaling of decentralised training [39, 58, 60, 65, 72, 86, 89, 93, 96, 102, 103, 112]. Ideally, the mission statement of a medical school had to reflect its clear intention to provide relevant training away from the large teaching hospital and explain to what end [26, 28, 106, 107]. Courageous and innovative solutions are necessary to achieve this ideal [96, 113]. In a subset of the leadership theme, some articles pointed to the need for local on-site leadership committed to the cause and engagement of role models and mentors for students [23, 37, 45, 73, 89–91, 114]. These local ‘champions’ were effective because they are familiar with the particular context and include clinical teachers and local physicians who are dedicated to training the next generation of health workers [23, 90].

#### Stakeholder engagement

In addition to community partnerships mentioned in the previous theme, engagement with multiple stakeholders such as the health services and medical school departments was seen as important [30, 36, 60, 72, 93, 96, 103]. Functioning partnerships were described as central to the successful implementation of decentralised training [83, 96]. Constant attention should be given to developing good relationships with stakeholders [60, 65, 69, 89] with the need to establish formal agreements, for example with the health services and communities [102]. The development of good relationships over time among the university, the facility, students, staff and supervisors facilitated collaborative and effective learning [39, 44, 55, 89].

#### Funding

Some studies pointed out that financial resources were key to the sustainability of decentralised training [38, 48, 82, 115]. Adequate funding for decentralised training is a concern emerging from the review. Decentralised training programmes were found to be expensive, and multiple sources of funding were needed, including government support [29, 48, 59, 83, 96, 106, 116, 117]. The need for student and faculty support at remote sites contributed to high costs [87]. Although we also searched for costing or funding models for decentralised training, we could not find anything specifically applied to preservice decentralised clinical training. Further research is needed as to the costs of decentralised training in comparison with the costs of the traditional model, taking into account the diversity of existing models [113, 118].

#### Evaluation

The reviewed articles indicated that it was important that decentralised training initiatives be evaluated both for programme feedback and development as well as building an evidence base of effective strategies [74, 75, 83, 93, 96, 104, 119]. Evaluation was more important in these programmes as they took place away from the main academic complex, where there was sometimes less control and structure [22]. In addition, evaluation provided the opportunity to compare the effectiveness of a diversity of models [87].

## Discussion

Some of what has emerged from this study was not unexpected. In the introduction to this article, for example, we already referred to the potential of decentralised exposure to enhance students’ learning experiences and to the need for commitment of all stakeholders to the success of establishing a decentralised site by providing the required resources. The value of the review lies in the way in which this message was consistently reaffirmed across multiple studies, thus strengthening the trustworthiness of the claims being made. Some findings, particularly those relating to student perceptions and the experience of supervisors, resonate with those described in an earlier systematic review of student learning in underserved areas [1].

Our intention with this scoping review was to determine the current status of decentralised training in the health professions as it is represented in the literature with a view to using our findings to support the development of a model for such training. We have not formally appraised the quality of the articles, rather generating a description of the decentralised training landscape and the factors that might enable or constrain it – this in keeping with our understanding of the aim of conducting a scoping review. Using the term ‘model’ is, however, a flat description and unintentionally suggests a set of criteria that, if in place, will enable the successful establishment and implementation of a decentralised training site. It belies the multi-layered complexity of the approach, which instead understands that the different themes that we have identified are interconnected, even interwoven, resulting in a series of unique, site-specific realities. This realisation has challenged us to reflect critically on our overarching goal of establishing a model and rather to consider the potential of a matrix approach that acknowledges the relationships that exist among the different components within each unique system.

Against this background, a number of implications for practice can be identified from this study. Firstly, Hirsh et al. [120] assertion, in 2007, that in terms of the student learning experience, ‘continuity’ (of care, curriculum and supervision) is a sound ‘organising principle’ for the clinical training of medical students and that such continuity is found in placements that challenge traditional approaches, remains valid. Linked to this is the influence of the ‘different’ (nontertiary) context that provides authentic, relevant learning experiences that have particular relevance for dealing with the burden of disease and the challenges linked to primary and secondary care in most LMICs. It reaffirms the drive for longer and more integrated offerings introduced by the early adopters of decentralised training [121–124] and further builds on the growing evidence that students’ academic outcomes are not disadvantaged when they move away from the academic complex [13]. Thus, an underlying theme speaks to ongoing calls for curriculum renewal [7], aligning student outcomes and learning opportunities to context and public need [125]. Curriculum adaptation should include the adoption of unique, contextualised assessment practices which are appropriately standardised and visibly quality assured [16]. We do, however, also recognise that there are inherent counter-forces in responding to the calls for curriculum renewal, even to calls for implementing decentralised training itself. Change can potentially tilt the balance of power that currently resides in the tertiary hospital and with specialist clinicians. Resistance to such change will need to be both recognised and managed.

A second implication for practice is that it takes a community to raise a doctor. Here community is seen in its broadest sense, including all stakeholders – facility (whether formal health care facilities such as a hospital or clinic or informal facilities common in many communities in LMICs), health practitioners, patients and people who live in the vicinity. This community also critically includes those who represent the sending institution and who have a responsibility in terms of ensuring an enabling learning environment. It should be noted, however, that the bias in terms of studies from developed countries meant that there was an implicit assumption that there would be sufficient resources to set up infrastructure at a decentralised site should such infrastructure not be in place. This is a problematic assumption both because many LMICs will not have such resources and because there is evidence of successful student placements even in severely underresourced contexts [1].

Thirdly, this review reminds us of the importance of leaders with vision, agents of change who are prepared to seek innovative and socially accountable solutions to the prevailing realities through challenging dominant thinking and developing meaningful relationships across multiple platforms. While the concept of decentralised learning is steadily becoming institutionalised, the challenges involved in developing and maintaining such programmes, especially in resource-constrained environments, imply that enthusiastic drivers within faculties and/or health services remain necessary.

Finally, there is, in fact, a golden thread seamlessly linking all of the themes, which is that of relationships between students and their supervisors, students and their patients, students and the community, the community and the facility, the community and the institution and so forth. These relationships include both the critical formal relationships amongst stakeholders, which are essential to the concept of symbiosis as a basis for quality medical education [126], and the deep interpersonal relationships arising from ongoing interactions amongst role-players in a decentralised clinical environment; the latter are more serendipitous and difficult to define and thus more challenging to achieve, yet essential in reaching the expected outcomes of decentralised student training. It is particularly important to foster such relationships in the complicated health systems that characterise many LMICs, where competing public and private systems may fail to deliver adequate health services to underserved populations in both urban and rural settings, with resultant limited capacity to support medical education. In addition, both the sending institution and hosting facility may struggle to identify community leadership, thus requiring their diplomacy, persistence and dedication in pursuing appropriate symbiosis.

This review had a number of limitations. Though we sought to focus on LMICs, the majority of the included studies, as was expected, reported data from developed countries (Australia (*n* = 36), the USA (*n* = 21) and Canada (*n* = 10)). The fact that only English publications were included in our study, therefore excluding work from non-English-speaking LMICs, should be regarded as a limitation. In addition, our definition of ‘decentralised’ included all training sites removed from the tertiary hospital and thus more than the rural context described in the majority of studies. We acknowledge that ‘decentralised training’ is a suitcase term for a suite of training models and approaches that are being implemented globally and that we may have missed out on studies that are potentially relevant but differently described. This has implications when considering the relevance of what we have found for our ongoing work. More work in this area, such as the recently published typology of longitudinal integrated clerkships [3], is needed. Although we recognise that evaluating educational outcomes are notoriously challenging, this study identifies a gap in this kind of evaluation in LMIC contexts, as well as inadequate exploration of outcomes in stakeholders other than students.

## Conclusions

Training students in a clinical environment is central to a twenty-first-century medical curriculum. Providing students with exposure to a range of clinical environments, including those outside of the academic tertiary hospital, across the training period has been shown to have value for the student. In addition, there is a growing body of literature that identifies the benefits of decentralised clinical training that extend beyond the value for the students’ learning experience to include benefits for other stakeholders and role-players. This review has sought to draw together current scholarship in the field to better understand the factors that characterise current approaches to decentralised training and its influence on the communities within which the training occurs. Evident from our findings are the multiplicity of such factors and their interconnectedness. Even as we have offered a representation of the student experience as nested within a particular context that is itself framed by the leadership and governance that direct it, we acknowledge the interplay amongst multiple identified factors across the different levels. Thus, each decentralised site is seen to have its own dynamic that may foreground certain elements and therefore may respond differently to the challenge of enabling student learning. This in turn influences the student experience. Going forward, these insights together with the knowledge base that has been established through this review have relevance in informing the further expansion of decentralised clinical training, including in LMIC contexts.

## Abbreviations

CDC: Centers for disease control and prevention
FMHS: Faculty of Medicine and Health Sciences
LMICs: Lower and middle-income countries
SU: Stellenbosch University
SUCCEED: Stellenbosch University Collaborative Capacity Enhancement through Engagement with Districts
UK: United Kingdom
USA: United States of America
WHO: World Health Organization
